# Cardiovascular Interactions between Fibroblast Growth Factor-23 and Angiotensin II

**DOI:** 10.1038/s41598-018-30098-1

**Published:** 2018-08-17

**Authors:** Min Pi, Ruisong Ye, Xiaobin Han, Benjamin Armstrong, Xue Liu, Yuanjian Chen, Yao Sun, L. Darryl Quarles

**Affiliations:** 1 Division of Nephrology, Department of Medicine, University of Tennessee Health Science Center, Memphis, USA; 2 Division of Cardiovascular Diseases, Department of Medicine, University of Tennessee Health Science Center, Memphis, USA

## Abstract

Both the activation of the renin angiotensin aldosterone system (RAAS) and elevations of circulating Fibroblast Growth Factor-23 (FGF-23) have been implicated in the pathogenesis of left ventricular hypertrophy (LVH) in chronic kidney disease. To investigate potential cross-talk between RAAS and FGF-23, we administered angiotensin II (Ang II) to wild-type rodents and the *Hyp* mouse model of excess FGF-23. Ang II administration for four weeks to wild-type rodents resulted in significant increases in systolic blood pressure and LVH. Unexpectedly, FGF-23 circulating levels were increased by 1.5–1.7 fold in Ang II treated animals. In addition, Ang II treatment increased expression of FGF-23 message levels in bone, the predominant tissue for FGF-23 production, and induced expression of FGF-23 and its co-receptor α-Klotho in the heart, which normally does not express FGF-23 or α-Klotho in physiologically relevant levels. *Hyp* mice with elevated FGF-23 exhibited increased blood pressure and LVH at baseline. Ang II administration to *Hyp* mice resulted further increments in blood pressure and left ventricular hypertrophy, consistent with additive cardiovascular effects. These findings suggest that FGF-23 may participate in unexpected systemic and paracrine networks regulating hemodynamic and myocardial responses.

## Introduction

Patients with chronic kidney disease (CKD) and end stage renal disease (ESRD) have high cardiovascular mortality associated with non-traditional risks factors^1–3^. Fibroblast growth factor-23 (FGF-23) has emerged as one of the most powerful predictors of adverse outcomes in these patients^4–7^. FGF-23 is a bone-derived hormone that regulates phosphate and 1,25(OH)_2_D metabolism through activation of the canonical FGF-23 receptor binary complex created by FGFR 1, 3 and 4 binding with α-Klotho (α-Kl), a type I membrane, β-glycosidase-like protein^8–12^. Progressive increases in circulating FGF-23 concentrations occur during the course of CKD, achieving levels that are several hundred times the normal range in advanced CKD and ESRD^7,13^. Elevations of FGF-23 are associated with adverse cardiovascular events and death. These adverse outcomes are attributed to effects of FGF-23 to stimulate left ventricular hypertrophy (LVH)^14–16^, and occur with small increments in circulating FGF-23 concentrations^17,18^.

The mechanism (s) whereby FGF-23 causes LVH uncertain, and multiple mechanisms have been proposed^19^. The leading hypothesis is that FGF-23 directly effects the heart to cause LVH through activation of FGFR4/PLCγ-dependent signaling in the myocardium^16,20,21^. This non-canonical, α-Kl-independent signaling pathway is controversial, because the tissue selectivity of FGF-23 is imparted by co-expression of α-Kl with FGFRs^8^, which is not expressed at physiological levels in the normal heart^22^. Alternatively, there are many kidney effects of FGF-23 mediated by activation of FGFRs/α-Kl in renal tubules that could lead to LVH and adverse cardiovascular outcomes. For example, FGF-23 may activate the renin-angiotensin-aldosterone system (RAAS), which is linked to a multitude of pathologic processes, including left ventricular hypertrophy, through suppression of 1,25(OH)_2_D, which would increase renin expression^23^. FGF-23 also reduces angiotensin converting enzyme 2 (ACE2) expression^24^, an enzyme which cleaves angiotensin II (Ang II) to generate vasodilatory angiotensin 1–7 peptides. RAAS activation and ACE2 insufficiency have been linked to cardiac hypertrophy and myocardial fibrosis^25^ and oxidative stress and inflammation^26^. In addition, FGF-23 administration to mice induces hypertension and LVH through stimulation of renal distal tubule sodium transport^27^. Finally, both FGF-23 suppresses kidney expression of α-Klotho^24^. α-Kl deficiency is linked to uremic cardiomyopathy through FGF-23 independent mechanisms^28^. Conversely, soluble Klotho (s-Kl) released into the circulation from ectodomain shedding is reported to exert cardioprotective effects^29^.

In the current study, we examined the cardiovascular interactions between Ang II and FGF-23. We found that Ang II administration to rodents causes LVH, increased circulating FGF-23 levels and ectopic expression of *Fgf-23* and *α-Klotho* in the heart, whereas Ang II administration to *Hyp* mice with preexisting elevation of FGF-23 levels resulted in additive effects on blood pressure and LVH. These observations provide a new conceptual framework for understanding the role of FGF-23 in adverse cardiovascular outcomes.

## Results

### Angiotensin II-induces hypertension, cardiac hypertrophy and increased FGF-23 expression in rodents

**Figure 1:**
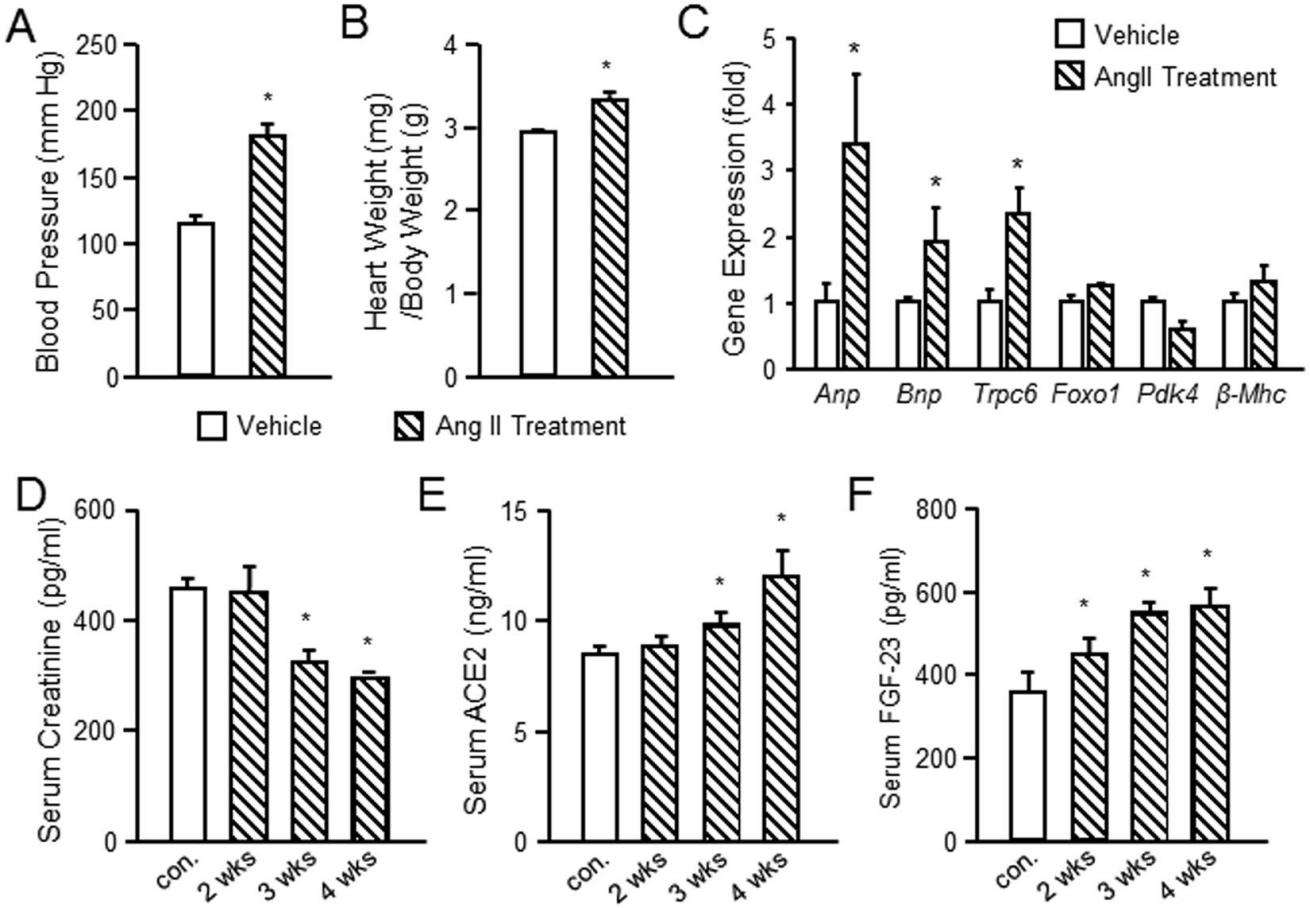
Ang II-induced hypertension and LVH in rats. (**A**) Blood pressure in Ang II treated and vehicle treated rats. (**B**) Cardiac hypertrophy as measured by heart weight/ body weight ratio in Ang II treated and vehicle treated rats. (**C**) Ang II increased the expression of the genes related to hypertrophy in the rat heart. (**D**) Serum concentrations of creatinine (**D**), ACE2 (**E**) and FGF-23 (**F**) in Ang II and vehicle treated rats. Animals treated with Ang II (35 μg/kg/h by minipump) or vehicle (saline) infusion for four weeks (n = 5/group). *p < 0.05 vs vehicle treated controls. All values are shown as mean ± SEM.

Ang II infusion is an established method for inducing hypertension and cardiac hypertrophy in rodents^30,31^. Consistent with prior reports, Ang II administered by osmotic minipump for 4 weeks (n = 5 per group) resulted in significant increases in systolic blood pressure, from 115 ± 5.5 to 182 9.5 in rats (Fig. 1A). The heart-weight-to-body-weight ratio (HW/BW, mg/g) was 3.35 ± 0.08 in Ang II treated rats compared to 2.93 ± 0.03 in vehicle treated controls (Fig. 1B). In addition, Ang II increased the expression of the genes related to hypertrophy^14,15,32^, including *Anp, Bnp* and *Trpc6, but not β-Mhc* in rat hearts, as well as *Foxo1* and *Pdk4*, factors regulating glucose oxidation in the heart^33,34^. (Fig. 1C). We found that serum creatinine was significantly lower after 3 and 4 weeks of Ang II treatment compared to controls (Fig. 1D), possibly related to pressure-mediated hyperfiltration. Ang II treated rats also exhibited a significant increase serum ACE2 levels after 4 weeks (Fig. 1D), suggesting that counter regulatory pathways were activated. Unexpectedly, intact serum FGF-23 levels were significantly increased in Ang II treated rats, reaching levels of 564 pg/ml by the 4^th^ week of treatment (Fig. 1F).

**Figure 2:**
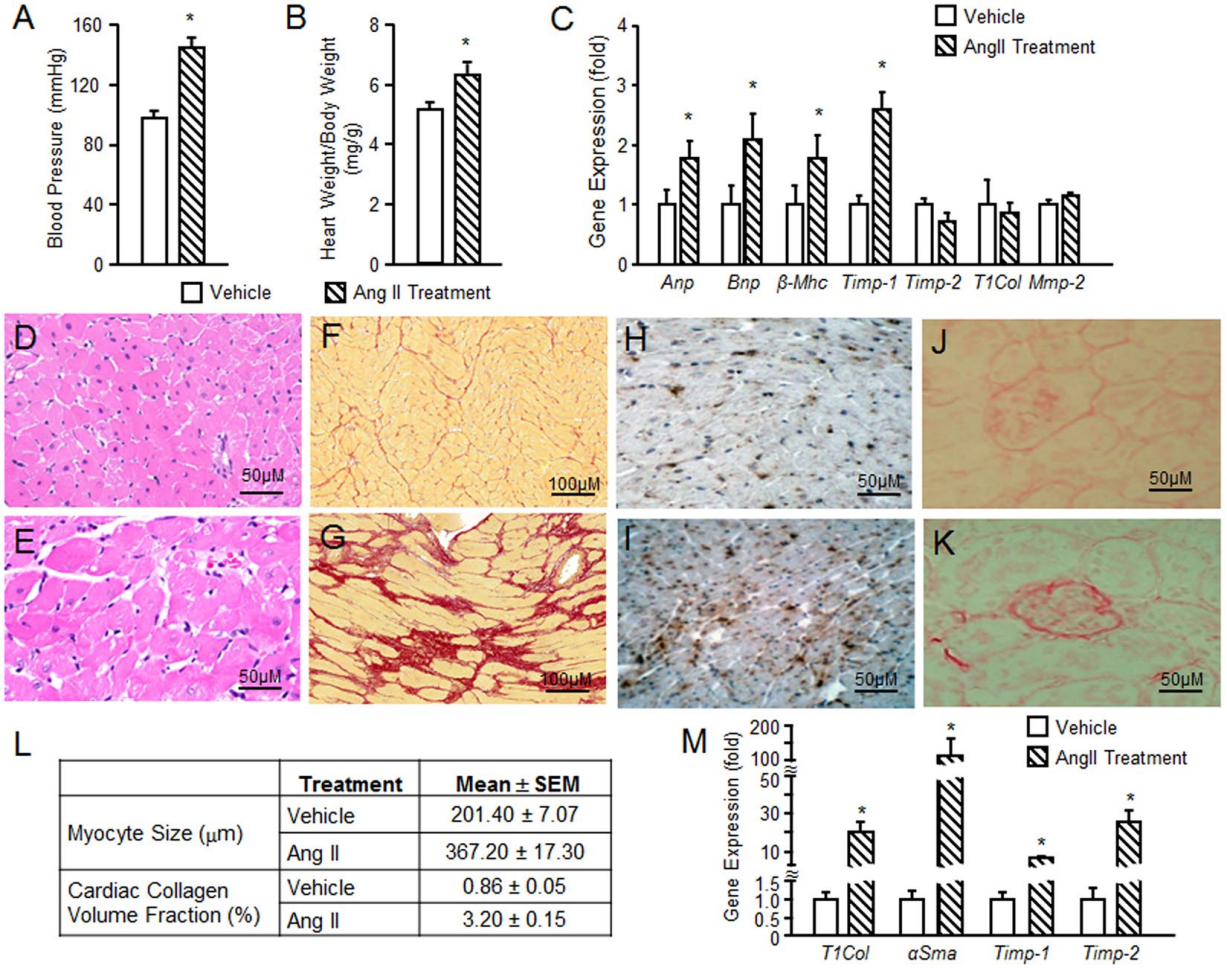
Ang II induced hypertension and LVH in mice. (**A**) Blood pressure in Ang II treated and vehicle treated mice. (**B**) Cardiac hypertrophy as measured by heart weight/ body weight ratio in Ang II treated and vehicle treated mice. (**C**) Ang II increased serum FGF-23 levels in Mice. (**D**) Ang II increased the expression of the genes related to hypertrophy in the mouse heart. (**E** and **F**) H&E staining of heart hypertrophy in Ang II treated mice (**F**) compared to control mice (**E**). (**F** and **H**) Mouse fibrotic responses in response to 4 weeks of Ang II infusion. Increased interstitial fibrosis in Ang II treated mice (**H**) compared to vehicle treated mice (**G**) by picrosirius red (PSR) staining. (**I**,**J**) Mouse cardiac inflammatory in response to 4 weeks of Ang II infusion. ED-1 staining of the normal myocardium of mouse (**I**). ED-1 (is the most widely used monoclonal antibody clone directed against the CD68 protein) marker of positive macrophages are accumulated in the damaged myocardium (**J**). (**K** and **L**) Ang II-induced kidney injure in mice. PSR straining in normal mouse kidney (**K**) and the kidney from Ang II treated mice showing glomerular sclerosis (**L**). Magnification X200. (**M**) Comparison of myocyte size and cardiac collagen volume fraction in Ang II treated and vehicle treated mice. Myocyte size was assessed on 5 μm cross-sectional hematoxylin-eosin stained slices. The outer border of transverse sectioned myocytes was drawn and myocyte area was calculated using NIH Image J software. (**N**) Comparison of fibrosis related gene expression in kidney from Ang II treated and vehicle treated mice. Animals treated with Ang II (35 μg/kg/h by minipump) or vehicle (saline) infusion for four weeks.n = 4/group, *p < 0.05 vs vehicle treated controls. All values are shown as mean ± SEM.

Ang II treatment, which is known to induce hypertension, significantly increased systolic blood pressure, from 98 ± 5 to 145 ± 7 mmHg in mice (Fig. 2A). The HW/BW ratio was 6.4 ± 0.4 in Ang II treated mice compared to 5.2 ± 0.2 in vehicle treated controls (Fig. 2B). The circulating FGF-23 level (pg/ml) increased from 185.5 ± 32.2 in vehicle treated mice to 321.8 ± 14.2 in Ang II treated mice (Fig. 2C). *Anp, Bnp, β-Mhc, and Timp-1 were* significantly increased in hearts from mice treated with Ang II compared to vehicle treated mice (Fig. 2D). Histological sections of the heart demonstrated hypertrophy of cardiomyocytes after Ang II administration (Fig. 2E,F). Using collagen-specific picrosirius red (PSR) staining for fibrosis, we observed minimal amount of collagen in the normal mouse heart (Fig. 2G), but cardiac interstitial fibrosis in the mice receiving Ang II infusion (Fig. 2H). Macrophages, staining positive for an ED-1 monoclonal antibody, were increased in myocardium in Ang II treated compared to vehicle treated mice (Fig. 2I,J). We found that Ang II infusion also resulted in glomerular sclerosis (Fig. 2K,L) in the kidney. In Fig. 2M, the myocyte size and cardiac collagen volume fraction were significantly increased 82% and 272% fold, respectively, in 4 weeks Ang II infusion mice compared to vehicle treated mice. We also found that *T1Col, αSma, Timp-1* and *Timp-2* were significantly increased in kidney of the mice receiving Ang II infusion compared to vehicle treated mice (Fig. 2N), consistent with the presence of renal fibrosis. Overall our findings are consistent with known effects of Ang II to cause LVH, cardiomyocyte hypertrophy and cardiac and kidney fibrosis^35–38^.

**Figure 3:**
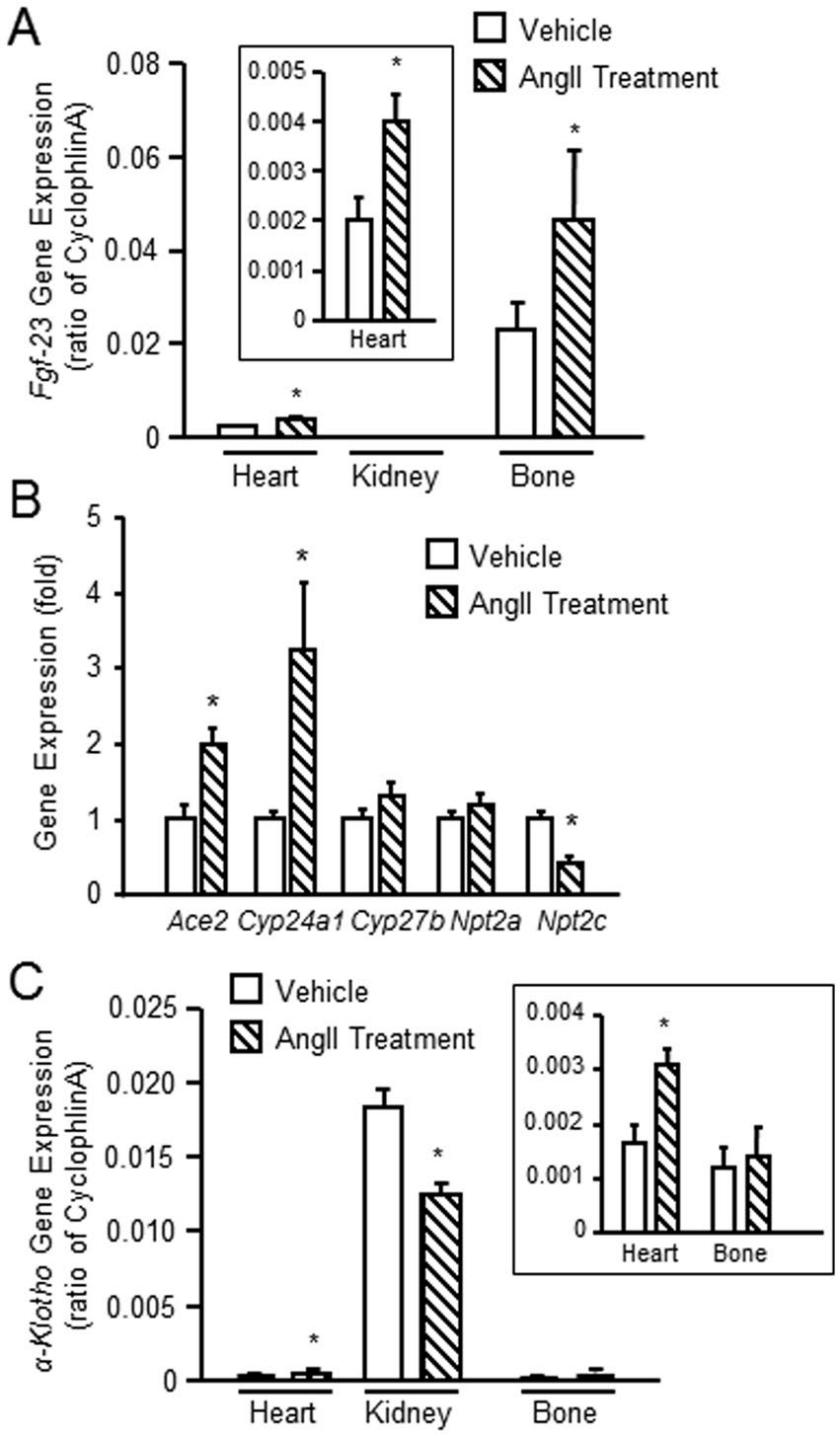
Ang II induced gene expression in heart, bone and kidney. (**A**) Comparison of *Fgf-23* message expression in heart, kidney and bone (without marrow) from Ang II or vehicle treated mice. (**B**) Comparison of FGF-23 message expression in the kidney from rats with/without Ang II treatment. (**C**) Comparison of *α-Kl* expression in heart, kidney and bone (without marrow) from Ang II treated with vehicle treated mice. n = 4/group, *p < 0.05 vs vehicle treated controls. All values are shown as mean ± SEM.

Consistent with bone as a possible source for the increased circulating FGF-23, we found that *Fgf-23* message expression was present in bone devoid of marrow of control mice, and was significantly increased in mouse bone after Ang II treatment (Fig. 3A). Efforts to test the effect of Ang II to directly stimulate FGF-23 production produced variable results (Supplemental Fig. 1); thus we are not able to determine if Ang II is regulating FGF-23 transcription in osteoblasts directly or through indirect effects mediated by Ang II effects on the kidney or other tissues, such as stimulating aldosterone-mediated increase in FGF-23^39^. Consistent with previous reports^40^, *Fgf-23* was predominately expressed in bone, but was detected at very low levels in the heart, but and undetectable in the kidney of control mice. We detected significant increases in *Fgf-23* message expression in the bone, but not kidney, of Ang II treated mice (Fig. 3A). We also observed increased expression of FGF-23 in the heart, but the increase was much lower than in bone, where FGF-23 is normally produced. We found that Ang II treated rats also had increments in *Cyp24a1* and decrements in *Npt2c* and *α-Kl* in the kidney (Fig. 3B), consistent with increased FGF-23 effects on the kidney. *Ace2* message expression was increased in the kidneys from Ang II treated rats, possibly due to effects of Ang II to increase this compensatory pathway and overriding effects of FGF-23 to suppress ACE2 (Fig. 3B). Interestingly, Ang II increased *α-Kl* message expression in the heart of mice, although the magnitude of *α-Kl* expression was low compared to the kidney, the predominate tissue that expresses *α-Kl* (Fig. 3C). The ectopic expression of FGF-23 along with *α-Kl* and the ubiquitous presence of FGFRs, suggests that Ang II may create a tissue environment for paracrine FGF-23 effects.

### Elevated FGF-23 is associated hypertension and cardiac hypertrophy in Hyp mice

**Figure 4:**
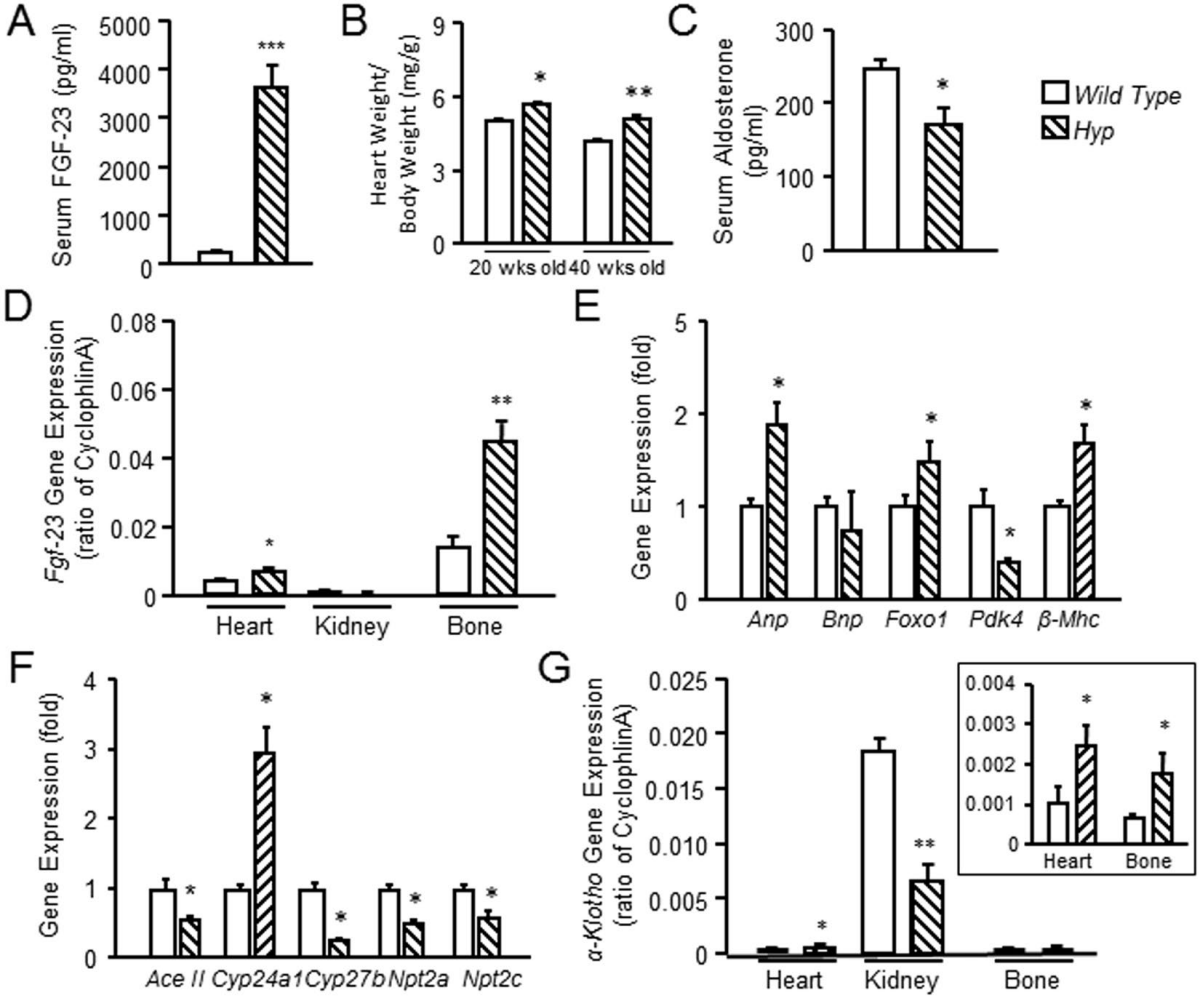
Increased FGF-23 is associated with LVH in *Hyp* mice. Comparison of serum FGF-23 levels (**A**), cardiac hypertrophy (**B**), and serum aldosterone levels (**C**) in *Hyp* and wild type mice at age of 20 or 40 weeks old. (**D**) Comparison of *Fgf-23* expression in heart, kidney and bone (without marrow) from *Hyp* and wild type mice at age of 20 weeks old. (**E**) Comparison of gene expression markers of cardiac hypertrophy in heart from *Hyp* and wild type mice at age of 20 weeks old. (**F**) Comparison of FGF-23 regulated genes expression in the kidney from *Hyp* and wild type mice at age of 20 weeks old. (**G**) Comparison of *α-Kl* expression in heart, kidney and bone (without marrow) from *Hyp* and wild type mice at age of 20 weeks old. n = 4/group, *p < 0.05 vs vehicle treated controls. All values are shown as mean ± SEM.

Primary elevations of FGF-23 are associated with LVH in the X-linked hypophosphatemic rickets (*Hyp*) mouse model of X-linked hypophosphatemic rickets^27^. *Hyp* mice had markedly elevated circulating FGF-23 levels (Fig. 4A), that were 6-fold greater than the elevations observed in Ang II treated animals (Fig. 1F). Similar to prior reports, the heart weight in 20 and 40 week-old *Hyp* mice was increased by 15% and 22% compared to controls, and serum aldosterone significantly suppressed due to FGF-23 effects to increase renal sodium reabsorption (Fig. 4B,C). FGF-23 is predominately expressed in osteoblasts and osteocytes in bone^41^. We also found that *Hyp* mice, which have elevated FGF-23 production due to inactivating *Phex* mutations in bone, had increased *Fgf-23* message expression in bone (Fig. 4D). Interestingly, however, *Hyp* mice also exhibited measurable and significantly increased FGF-23 message levels in heart compared to wild-type controls (Fig. 4D). Gene expression markers of cardiac hypertrophy, including *Anp, Foxo1*, and *β-Mhc*, but not *Bnp* and *Ddk4*, were also increased in the heart of *Hyp* mice (Fig. 4E). The renal gene expression profile in the kidney of *Hyp* mice was consistent with known actions of FGF-23 to suppress *Ace2, Cyp27b1, Npt2a, Np2tc* and increase in *Cyp24a1* (Fig. 4F). In addition, α*-Kl* expression was decreased in the *Hyp* kidney, but α*-Kl* expression was slightly but significantly increased in the heart of *Hyp* mice (Fig. 4G).

### Ang II and FGF-23 exhibit additive effects on hypertension and cardiomegaly in mice

**Figure 5:**
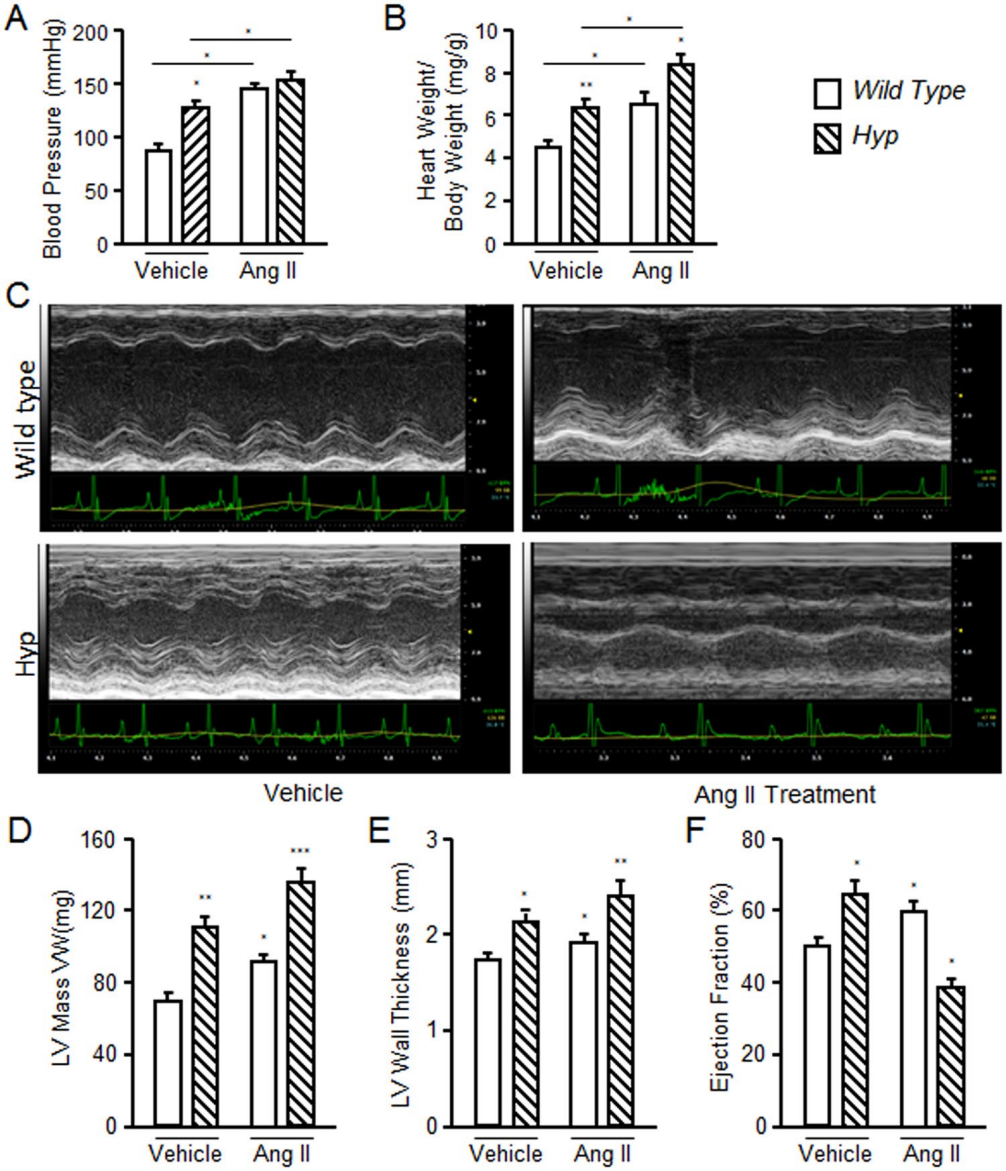
Effect of Ang II on blood pressure and LVH in WT and *Hyp* mice. Four-week-old wild-type and *Hyp* mice were treated with Ang II for 4 weeks. Blood pressure, echocardiography and heart to body weight ratio were measured at the end of Ang II treatment. *Hyp* mice showed elevated blood pressure in the absence of Ang II treatment and blood pressure in *Hyp* mice was significantly increased by treatment of Ang II compared to control *Hyp* mice. Ang II treatment in WT mice increased blood pressure (**A**) and induced LVH (**B**). Untreated *Hyp* mice exhibited LVH and Ang II administration resulted in worsening of LVH in *Hyp* mice, as assessed by ratio of heart weight to body weight (**B**) and echocardiography (**C**). (**D** and **E**) *Hyp* mice showed higher LV mass and LV wall thickness, which were further increased after Ang II administration. (**F**) Ang II increased ejection fraction in WT mice and decreased EF in *Hyp* mice compared to wild type mice. Animals treated with Ang II (35 μg /kg/h by minipump) or vehicle (saline) infusion for four weeks. n = 4/group, *p < 0.05 and **p < 0.01 vs vehicle treated controls. All values are shown as mean ± SEM.

To gain insights into the potential contribution of elevated FGF-23 to LVH observed in Ang II treated animals, we compared FGF-23 levels and the severity of LVH between *Hyp* and Ang II treated wild-type mice. We administered Ang II to *Hyp* mice to determine if the cardiovascular effects of FGF-23 and Ang II are additive. Eight week-old wild-type and *Hyp* mice were treated with 35 μg/kg/h Ang II by implanted minipump for 4 weeks. At baseline, systolic blood pressure (BP) were significantly higher in *Hyp* compared to in wild-type mice (Fig. 5A). Ang II administration further increased blood pressure in *Hyp* mice by ~17%, from a mean systolic blood pressure of 126.8 ± 3.4 to 148 ± 2.1 mmHg (Fig. 5A). In contrast, Ang II treatment of wild-type mice increased systolic blood pressure by ~ 63%, from 89.5 ± 1.9 to 145.8 ± 3.7 mmHg. The maximum blood pressure level induced by Ang II was not different between *Hyp* and wild-type mice. LVH was also present in *Hyp* mice at baseline compared to wild-type mice by HW/BW and echocardiographic parameters. Ang II increased HW/BW ratio in both *Hyp* and wild-type mice (~32% and ~48%, respectively) (Fig. 5B). Echocardiographs showed that *Hyp* mice had a greater left ventricular wall thickness at baseline compared to wild-type mice. Ang II elevated left ventricular wall thickness in wild-type mice, and resulted in additive effects to further increase LVH in *Hyp* mice. (Fig. 5C–F).

## Discussion

Why FGF-23, a hormone that regulates mineral homeostasis, has hemodynamic effects is a physiological enigma. These studies provide a potential explanation by showing for the first time that Ang II administration increases circulating FGF-23 levels in animal models *in vivo*. Treatment of rodents with Ang II stimulated the expression of FGF-23 message in bone, the physiological site FGF-23 production^40,42^, as well as the ectopic expression of FGF-23 in the heart, which does not normally express FGF-23 in physiological amounts^40^. Similar to RAAS activation, the sympathetic nervous system (SNS) has recently been shown to stimulate FGF-23 production in bone through β-adrenergic signaling pathways^43,44^. Thus, activation of RAAS and SNS, which are key hemodynamic regulators associated with adverse outcomes in CKD^45–47^, are important regulators of FGF-23.

Elevations of FGF-23 are purported to contribute to adverse cardiovascular effects as evidence by the finding of hypertension and LVH in the *Hyp* mouse model of FGF-23 excess^24,27^. We also have found that *β-Mhc* expression was significantly increased in heart from *Hyp* mice (Fig. 4E), which is similar to findings from *Dmp1* knockout mice, another hereditary model of excess FGF-23^48^. Moreover, the observation that elevations of FGF-23 exacerbate the severity of Ang II-induced hypertension and LVH in the *Hyp* mouse model, indicates that FGF-23 and RAAS may work through complementary pathways to enhance cardiovascular responses. Finally, small increments in FGF-23, as observed in Ang II treated animals, are associated with adverse cardiovascular outcomes in clinical observation studies^49^. Thus, the regulation of FGF-23 by RAAS and the additive cardiovascular effects of FGF-23 and Ang II potentially identifies a novel feed-forward endocrine network to enhance hemodynamic responses and leads to the ectopic production of FGF-23 and α-Klotho to locally reconstitute canonical FGF-23/FGFR/α-Kl signal in the myocardium^7,15,19^.

The mechanism whereby Ang II stimulates FGF-23 expression is not defined by our studies. Increased FGF-23 might result from direct effects to activate AT1 receptors in osteoblasts^50^. Efforts to test effects of Ang II to stimulate FGF-23 production in osteoblasts, however, produced variable results (Supplemental Fig. 1). Alternatively, increased FGF-23 might occur secondary to Ang II stimulation of aldosterone and TNF-α production, both which can increase FGF-23^39,51^, or suppression of Klotho expression in the kidney, leading to end organ resistance and secondary elevations in FGF-23^52^. Finally, Ang II stimulation of FGF-23 may play a role in skeletal homeostasis. Ang II excess has been implicated in the development of osteoporosis; and Ang II inhibits bone mineralization through activation of AT1 receptors in osteoblasts^50,53–55^. In this context, Ang II regulation of FGF-23 and bone mineralization might represent another component of the bone-kidney endocrine axis whereby FGF-23 coordinates bone mineralization and renal phosphate handling. Future studies will be needed to understand the mechanisms whereby Ang II increases the systemic an local tissue production of FGF-23, and define its contribution to the cardiotoxic actions of excess SNS and RAAS stimulation^45^.

We also do not define the exact mechanisms whereby FGF-23 exacerbates the cardiovascular effects of Ang II. Ang II activation of AT1 receptors causes hypertension and cardiac hypertrophy in the heart^30^ and stimulates renal sodium reabsorption and suppresses α-Klotho expression in the kidney^56,57^. There are several possibilities. First, kidney-specific deletion of α-Klotho, an FGF-23 regulated gene, also causes salt-sensitive hypertension in mice^58^. Consequently, Ang II and FGF-23 additive effects on renal tubular functions regulating blood pressure and cardiomegaly may account for enhanced hemodynamic responses. In support of this possibility, FGF-23 activation of FGFR/α-Klotho complexes in renal tubules to stimulate distal tubular sodium reabsorption^27^, and suppress *Ace2* and *α-Klotho* expression^19,24^ may enhance Ang II hypertensive actions.

Alternatively, suppression of *Ace2* by FGF-23 might prolong the actions of Ang II and prevent its conversion to the vasodilatory Ang 1–7^59^. Interestingly, Ang II is reported to down-regulate ACE2 in the kidney and the heart^60^. However, we observed, possibly for the first time that circulating levels of ACE2 are increased in the circulation in response to Ang II treatment. This suggests that Ang II induces the ectodomain shedding of ACE2, and may account for the observed association between elevated circulating ACE2 and hypertension^61^.

Second, FGF-23 also suppresses 1,25(OH)_2_D, which is known to have hemodynamic effect through suppression of renin^23^. Finally, reduced Klotho may contribute to the cardiovascular effects of FGF-23, since sKlotho (sKL) released by ectodomain shedding of α-Klotho by the kidney has cardioprotective effects by downregulating TRPC6 channels in cardiomyocytes^28,62–65^, and administration of sKl inhibits RAS and normalizes blood pressure in mouse models of kidney diseases^66^.

Third, increased FGF-23, either systemically or locally, might act through the non-canonical direct activation of FGFRs in the heart by FGF-23 (*i.e*., α-Klotho independent effects)^16^ or through Ang II induction of ectopic expression of FGF-23 and α-Klotho in the heart and local reconstitute FGFR/α-Klotho signaling in the stressed heart. With regards to the latter, we found that Ang II administration increased FGF-23 and α-Klotho message levels in heart, a tissue that does not normally express FGF-23 or α-Klotho in physiological amounts. These findings are consistent with other studies showing ectopic expression of FGF-23 and α-Klotho in the heart under disease conditions^16,67^. Ang II administration to α-Kl transgenic mice, which overexpress α-Kl in the heart and other tissues, exacerbates LVH and cardiac fibrosis^68^, supports the notion that activation of ectopically expressed FGFR/α-Klotho complexes in the myocardium can lead to cardiac hypertrophy. Finally, FGF-23 could have direct effects on vascular smooth muscle and vascular calcification through Klotho-mediated nitric oxide synthesis and oxidative stress^69–72^.

FGF-23 is reported to be expressed in macrophages that do not normally express FGF-23 in response to stress and inflammation^73^. Ectopic expression of FGF-23 in macrophages and effects on cells in the myeloid lineage has been purported to regulate innate immune responses outcomes^67,73,74^. The AT1 receptor is expressed in immune cells and Ang II alters their inflammatory functions^75^. FGF-23 in activated macrophages infiltrating hypertrophic hearts may contribute to cardiac fibrosis and abnormal expression of FGF-23 in cardiomyocytes^67,16^. FGF-23 may also interact with innate immune responses through suppression of 1,25D production by the kidney^56,76,77^. Interestingly, in spite of seeing both cardiac and renal inflammation and fibrosis, we did not see ectopic expression of FGF-23 in the kidney in response to Ang II treatment. This contrasts to the expression of FGF-23 in the renal epithelium that is observed in polycystic kidneys that is thought to be induced by inflammation^52^.

Pharmacological blockade of FGF-23 or Ang II will be need to tease out the indirect effects of FGF-23 from those mediated by Ang II activation of angiotensin receptors^78^.The clinical importance of cross talk between RAAS and FGF-23, however, is suggested by the finding that FGF-23 levels are higher in patients with heart failure not treated with angiotensin converting enzyme inhibitors (ACEi) and patients in the top tertile of elevated serum FGF-23 exhibit a lower risk of adverse events after treatment with ACEi^79^. The Ang II and FGF-23 endocrine network may help explain why elevated FGF-23 is associated with enhanced responses to angiotensin-converting enzyme inhibitor (ACEi) therapy in patients with heart failure but without CKD^79,80^.

The presence of hypertension and LVH in patients with XLH and the *Hyp* mouse homologue of this disease is variably reported. Similar to our findings, Erben’s group found that *Hyp* mice have LVH due to FGF-23 dependent effects on sodium reabsorption in the kidney^81^, and patients with XLH are reported to have exercise induced increases in BP^82^. In contrast, several studies have failed to identify associations between elevated FGF-23 and HTN or LVH in *Hyp* and the *Dmp1* null mouse model of autosomal recessive hypophosphatemia^48,83–85^. The reasons for these differences are unknown, but likely reflect age, dietary, genetic and/or environmental modifiers.

**Figure 6:**
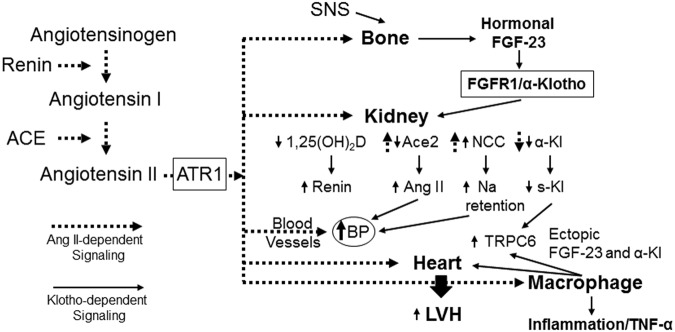
Crosstalk between RAAS and FGF-23 pathways. A new schema of hemodynamic regulation suggests actions of Ang II to stimulate FGF-23 in bone (afferent limb), leading to additive renal effects with Ang II (efferent limb) to increase blood pressure and cardiomegaly. In this model, FGF-23 effects are mediated by activation of FGFR/α-Klotho complexes, whereas Ang II activates AT1 receptors in renal tubules to enhance sodium transport, and to suppress α-Klotho. The resulting positive sodium balance leads to increased blood pressure and the reduced levels of soluble Klotho (s-Kl) may enhance cardiotoxicity through s-Kl-dependent TRPC6 cardiac effects. In contrast, FGF-23 suppresses, whereas Ang II stimulates *Ace2*, making degradation of Ang II and formation of Ang 1–7 a possible point of differential control. FGF-23 also suppresses 1,25(OH)_2_D synthesis, an effect predicted to increase renin production by the kidney. This novel feed-forward endocrine pathway may contribute to the association between elevated FGF-23 and adverse cardiovascular outcomes. In addition, Ang II upregulates FGF-23 and α-Klotho expression the heart and macrophages to possible reconstitute FGFR/α-Klotho signaling leading to paracrine effects of FGF-23 to induce cardiac hypertrophy and stimulate inflammation.

In summary, we propose a new schema for understanding cardiovascular homeostasis whereby Ang II stimulates the release of FGF-23 into the circulation and the ectopic expression of FGF-23 and its co-receptor α-Klotho in the heart; in turn, the systemic and local elevations of FGF-23 augment the cardiovascular effects of Ang II through multiple potential molecular mechanisms that include renal sodium absorption, suppression of 1,25(OH)_2_D_3_ and enhanced renin production, *Ace2* expression and systemic effects of α-Klotho (Fig. 6). If so, FGF-23 may participate in previously unrecognized systemic and local regulatory networks whereby the sympathetic nervous system and renin angiotensin system control hemodynamics^44^ and FGF-23 may link SNS and RAAS to inflammation and oxidative stress^67,86^, thus contributing to adverse effects in CKD and other conditions, such as congestive heart failure. Additional investigations are needed to understand the afferent pathways whereby Ang II stimulates FGF-23, the relative importance of the multiple efferent pathways potentially mediating FGF-23 associated cardiac toxicity, and the feedback pathway that shuts off this Ang II and FGF-23 feed forward loop.

## Methods

### Animals

Eight week old male SD rats and 4 month old male C57BL/6 J and male *Hyp* mice (C57BL/6-*Phex*^*Hyp-2J*^/J) were treated with Ang II at the dose of 35 μg/kg/h respectively given subcutaneously by implanted minipump^87,88^. Vehicle treated littermate rats and mice served as controls. The animals were fed with *ad lib* regular chow, Harlan Teklad 2018 (including 1% calcium, 0.7% phosphorus, 1.5 IU/g Vitamin D3; Harlan Teklad, Madison, WI, USA). Heart, kidney, and bone were collected after 4 weeks of Ang II infusion, the tissues were used for RNA purification, real time RT-PCR, histology and immunohistochemistry. Cardiac collagen volume was detected by picrosirius red (PSR) staining in cardiac sections and quantitated using a computer image analysis system (NIH image 1.6) as we previously reported^87^. Cardiac hypertrophy was assessed by heart-weight-to-body-weight ratio^89^.

This study was approved by the University of Tennessee Health Science Center Animal Care and Use Committee. The investigation conforms to the *Guide for the Care and Use of Laboratory Animals* published by the US National Institutes of Health.

### Blood pressure measurements

Blood pressure was measured by the tail-cuff plethysmography method in unanesthetized mice using a Hatteras Instruments SC1000 Blood Pressure Analysis System as previously described^61^. Measurements were made on the day at end of study 4 weeks after mini implantation.

### Histology and Immunohistochemistry

Mouse heart and kidney tissues were embedded into OCT compound (Tissue-Tek, Sakura Finetek USA; Torrance, CA, USA). Cryostat sections (6 μm) were air-dried, fixed in 10% buffered formalin for 5 min, and washed in phosphate-buffered saline (PBS) for 10 min. For H&E staining, the cryostat sections were rinsed in H_2_O, dipped into Mayer’s hematoxylin and agitated for 30 sec. Then the slide was rinsed in H_2_O for 1 min, and stained with 1% eosin Y solution for 10–30 sec, and dehydrated and mounted. Cardiac sections (6 m) were prepared to determine the fibrillar collagen accumulation by collagen-specific picrosirius red staining and observed by light microscopy as previously reported^38,90^. Collagen volume fraction of each section was determined using a computer image analyzing system (NIH image, 1.60), as previously reported^38,90^. Cardiac expression of ED-1 (a marker of macrophages) was detected by immunohistochemistry. Cryostat cardiac sections (6 μm) were incubated with a primary antibody against ED1 (Sigma, St. Louis, MO, USA) for 1 hour at room temperature. The sections were then incubated with the immunoglobulin G peroxidase–conjugated secondary antibody (Sigma) for 1 hour at room temperature and incubated with 0.5 mg/ml diaminobenzidine tetrahydrochloride 2-hydrate +0.05% hydrogen peroxide for 5 minutes. Negative control sections were incubated with the secondary antibody alone. All sections were counterstained with hematoxylin, dehydrated, mounted, and viewed by light microscopy^91^. We used Nikon-2 Optiphot-2 microscopy and at 20X objective lens. We analyzed 5 difference locations in per slide and total 3 slides per sample were analyzed.

### Real time RT-PCR

For quantitative real-time RT-PCR assessment of the markers of hypertrophy, fibrosis and *Fgf-23* expression (Supplemental Table 1), we isolated and reverse transcribed 2.0 µg of total RNA from the long bone, kidney and heart of mice with/without Ang II treatment as described previously^92^. PCR reactions contained 100 ng of template (cDNA or RNA), 300 nM each of forward and reverse primer, and 1X iQ SYBR Green Supermix (Bio-Rad, Hercules, CA, USA) in 50 µL. Samples were amplified for 40 cycles in an iCycler iQ Real-Time PCR Detection System (Bio-Rad) with an initial melt at 95 °C for 10 minutes, followed by 40 cycles of 95 °C for 15 seconds and 60 °C for 1 minute. PCR product accumulation was monitored at multiple points during each cycle by measuring the increase in fluorescence caused by the binding of SybrGreen I to dsDNA. The threshold cycle^93^ of tested-gene product from the indicated genotype was normalized to the *C*_*t*_ for *cyclophilin A*. Dissociation analysis was used to confirm the presence of a single transcript and lack of primer-dimer amplification in all PCR reactions.

### Serum Biochemical Measurements

Blood was collected using a retroorbital bleeding technique. Serum was separated by using Serum Separator Tubes (BD Life Sciences, Franklin Lakes, NJ, USA). Serum FGF-23 levels were measured using an FGF-23 ELISA kit (Kainos Laboratories, Inc., Tokyo, Japan). This kit is measurement for intact FGF-23. Serum creatinine was measured, using Creatinine Liquicolor Test (Stanbio Laboratory, Boerne, TX) as described previously^94^. Serum ACE2 and aldosterone were measured by using Angiotensin II Converting Enzyme (ACE2) WLIA Kit (San Diego, CA, USA) and Aldosterone EIA kit from Cayman chemical (Ann Arbor, MI, USA), respectively.

### Statistics

We evaluated differences between groups by one-way analysis of variance, followed by *a post-hoc* Tukey’s test. Significance was set at p < 0.05. All values are expressed as means ± SEM. All computations were performed using the Statgraphic statistical graphics system (STSC Inc.).

## Electronic supplementary material

Supplementary Information
