# Supplementary material for: Cardiovascular Interactions between Fibroblast Growth Factor-23 and Angiotensin II
Source: Sci Rep. 2018 Aug 17;8:12398. doi: 10.1038/s41598-018-30098-1 (PMC6098163; doi:10.1038/s41598-018-30098-1)
Supplement: Supplementary file 1 — Supplementary Information [file 41598_2018_30098_MOESM1_ESM.pdf]

**Cardiovascular Interactions between Fibroblast Growth Factor-23 and Angiotensin II**

Min Pi[1], Ruisong Ye[1], Xiaobin Han[1], Benjamin Armstrong[1], Xue Liu[2], Yuanjian Chen[2], Yao Sun[2], and L. Darryl Quarles[1*]

Division of Nephrology[1], Division of Cardiovascular Diseases[2],

Department of Medicine, University of Tennessee Health Science Center

## Supplemental Information

Although in ESRD increased serum FGF-23 correlates with serum phosphate levels and dietary phosphate loading increases FGF-23 in animal models, the regulation of FGF-23 is complex at least in part through possible effects of Ang II to directly stimulate FGF-23 message expression in osteoblasts, which express AT1 receptors [41]. AT1 receptors are present in ROS17/2.8 osteoblasts [41], and Ang II treatment resulted in a 2-fold increase in FGF-23 message expression in these cells (Supplemental Fig. 1A). However, we failed to observed effects of Ang II to stimulate FGF-23 protein secretion or FGF-23 promoter activity in MC3T3-E1 osteoblasts (Supplemental Fig 1B and C). Treatment of RAW 246.7 with lipopolysaccharide (LPS) and INF-γmacrophages to induce M1 macrophage activation significantly increased FGF-23 expression, but Ang II treatment did not stimulate FGF-23 in resting, M1, or M2 activated macrophages (Supplemental Fig 1D).

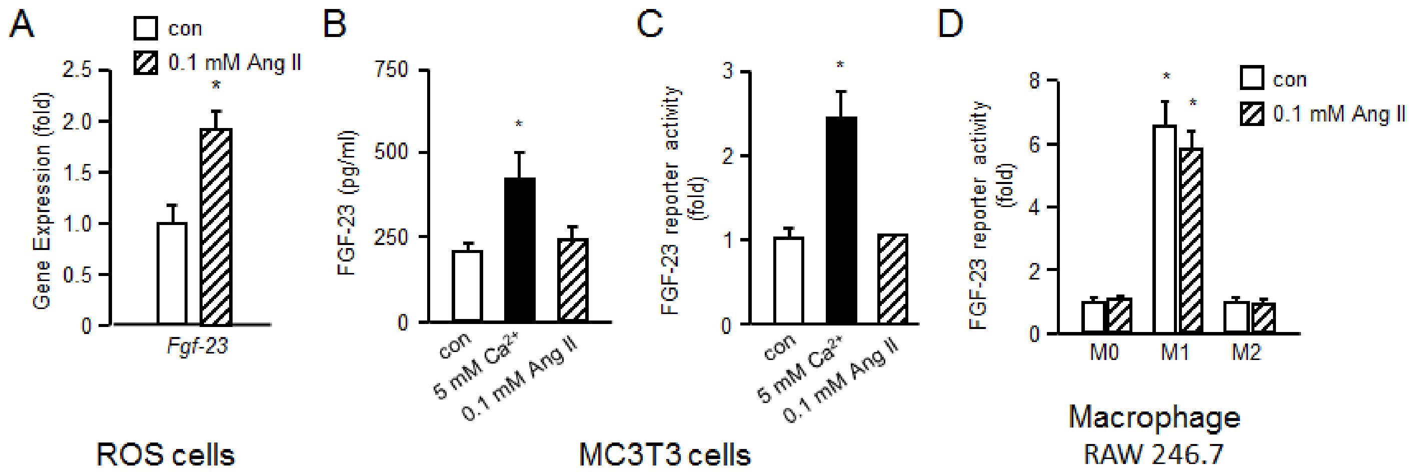

Supplemental Table 1. The nucleotide sequences of primer sets for real-time PCR in this study.

| Gene Name | Gene ID | Forward | Reverse |
|---|---|---|---|
| *rAnp* | NM_012612 | tttgctgaaggcaagaggtt | ttgtttctggctgcattctg |
| *rBnp* | NM_031545 | cagctcttgaaggaccaagg | aagagacccaggcagagtca |
| *rβ-MHC* | NM_ 017240 | tggcaccgtggactacaata | tacaggtgcatcagctccag |
| *ratFoxo1* | NM_001191846 | aaccagtccaactcgaccac | tgctcataaagtcggtgctg |
| *rPdk4* | NM_053551 | cctgtgatggacaattcacg | ctgccagtttctccttcgac |
| *rAce2* | NM_001012006 | gctaaacatgatggcccact | cccacagtcgaattcctgtt |
| *rCyp24a1* | NM_201635 | tgggtgaatacgctctaccc | tatccagcagagagccaggt |
| *rCyp27b1* | NM_053763 | acacctagcttcctggctga | agcgctctggacaatgactt |
| *rNpt2a* | NM_013030 | aacatcgggacctctgtcac | atgttgaaggaggcaaccac |
| *rNpt2c* | NM_139338 | agactgctctgccatcacct | gttaagcctgctccaacgag |
| *mAnp* | NM_008725 | cttcttcctcgtcttggcct | ctgcttcctcagtctgctca |
| *mBnp* | NM_008726 | catggatctcctgaaggtgc | ccttcaagagctgtctctgg |
| *mβ-MHC* | NM_080728 | aagcagcagttggatgagcg | cctcgatgcgtgcctgaagc |
| *mFoxo1* | NM_019739 | gcagccaggcatctcataa | cctaccatagccattgcagc |
| *mTrpc6* | BC141131 | cgctgccaccgtatgg | ccgccggtgagtcagt |
| *mMmp-2* | NM_008610.2 | acctgaacactttctatggctg | cttccgcatggtctcgatg |
| *mT1Col* | NM_007742 | gctcctcttaggggccact | attggggacccttaggccat |
| *mαSma* | X13297 | cccaactgggaccacatgg | tacatgcgggggacattgaag |
| *mTimp-1* | BC051260 | cgagaccaccttataccagcg | atgactggggtgtaggcgta |
| *mTimp-2* | M93954 | tcagagccaaagcagtgagc | gccgtgtagataaactcgatgtc |
| *mFgf-23* | NM_022657 | atgctagggacctgccttaga | ggagccaagcaatggggaa |
| *mα-Klotho* | NM_013823 | actacgttcaagtggacactact | gatggcagagaaatcaacacagt |
| *Cyclophilin A* | NM_008907 | ctgcactgccaagactgaat | ccacaatgttcatgccttct. |

Anp, natriuretic peptide A; Bnp, natriuretic peptide B; T1Col, collagen, type I, alpha 1; Cyp24a1, cytochrome P450 family 24 subfamily A member 1; Cyp27b1, cytochrome P450 family 27 subfamily B member 1; Ddk4, pyruvate dehydrogenase kinase, isozyme 4; Foxo 1, forkhead box O1; Npt2, solute carrier family 34 (sodium phosphate), member 1; β-Mhc, myosin, heavy polypeptide 7, cardiac muscle, beta; Mmp-2, matrix metallopeptidase 2; Phex, phosphate regulating endopeptidase homolog, X-linked; αSma, vascular smooth muscle alpha-actin; Timp-1, tissue inhibitor of metalloproteinase 1; Timp-2, tissue inhibitor of metalloproteinase 2; Trpc6, transient receptor potential cation channel subfamily C member 6.
